# Supplementary material for: Prognostic Factors of Early Stage Epithelial Ovarian Carcinoma
Source: Int J Environ Res Public Health. 2019 Feb 21;16(4):637. doi: 10.3390/ijerph16040637 (PMC6406698; doi:10.3390/ijerph16040637)
Supplement: Supplementary file 1 [file ijerph-16-00637-s001.pdf]

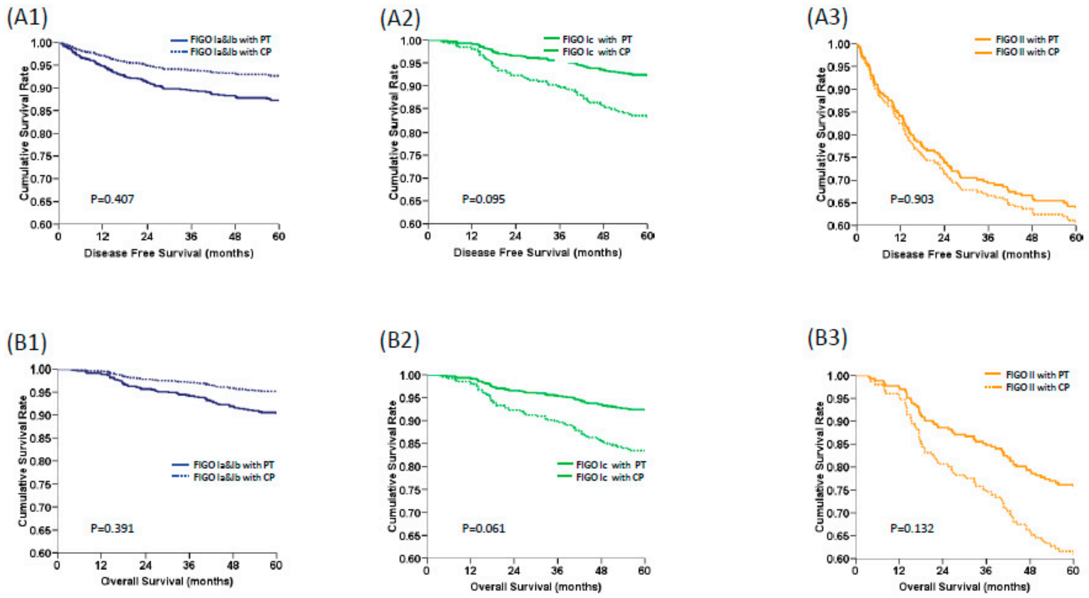

Supplementary Figure 1. Survival curves of 437 early stage epithelial ovarian carcinoma women stratified by chemotherapeutic regimens and FIGO stage in multivariate Cox regression models after adjusting for histologic types, tumor grade, and age at diagnosis. **(A)** Estimated 5-year disease-free survival (DFS) curves. A1: stages Ia/Ib, A2: stage Ic, A3: stage II. There was no difference in 5-year DFS between the platinum and paclitaxel (PT) or platinum and cyclophosphamide (CP) regimens for the different stages: stages Ia/Ib (87.1% vs. 92.5%, aHR=1.76, 95% CI=0.46-6.64 p=0.407), stage Ic (82.8% vs. 74.7%, aHR=0.62, 95% CI=0.36-1.08, p=0.095), stage II (63.8% vs. 60.7%, aHR=0.95, 95% CI= 0.46-1.99, p=0.903). **(B)** Estimated 5-year overall survival (OS) curves. B1: stages Ia/Ib, B2: stage Ic, B3: stage II. The 5-year OS of PT and CP regimens did not differ in all three stages: in stages Ia/Ib (90.4% vs. 95.0%, aHR=2.02, 95% CI=0.41-10.0, p=0.391), stage Ic (91.5% vs. 83.4%, aHR=0.49, 95% CI=0.23-1.03, p=0.061), and stage II (75.3% vs. 61.0%, aHR=0.56, 95% CI=0.27-1.19, p=0.132) (all by log-rank test).

Supplementary Table 1. Clinicopathologic characteristics of 437 early stage ovarian cancer women by hospitals.

**Characteristics**	**Total (*n* = 437)**		**NTUH (*n* = 248)**		**VGH-TP (*n* = 189)**	
	**Number**	**%**	**Number**	**%**	**Number**	**%**
Year of Diagnosis						
2000-2005	192	43.9	106	42.7	86	45.5
2006-2010	245	56.1	142	57.3	103	54.5
Age						
Mean (SD)	50.1 (11.0)		49.9 (11.2)		50. 4 (10.8)	
≤50	234	53.5	131	52.8	103	54.5
>50	203	46.5	117	47.2	86	45.5
FIGO stage						
Ia and Ib	101	23.1	41	16.5	60	31.7
Ic	244	55.8	165	66.5	79	41.8
II	92	21.1	42	16.9	50	26.5
Histology						
Serous	61	14.0	34	13.7	27	14.3
Mucinous	58	13.3	31	12.5	27	14.3
Endometrioid	119	27.2	63	25.4	56	29.6
Clear cell	164	37.5	102	41.1	62	32.8
Others *	35	8.0	18	7.3	17	9.0
Tumor grade						
I	88	20.1	75	30.2	13	6.9

II	102	23.3	26	10.5	76	40.2
III #	247	56.5	147	59.3	100	52.9
Adjuvant therapy						
PT	249	57.0	106	42.7	143	75.7
CP	188	43.0	142	57.3	46	24.3
Regimens and number of cycles						
PT (6)	192	43.9	88	35.5	104	55.0
PT (3-5)	57	13.0	18	7.3	39	20.6
CP (6)	137	31.4	107	43.1	30	15.9
CP (3-5)	51	11.7	35	14.1	16	8.5
Follow-up period mean (SD), months	85.9 (43.5)		86.3 (42.9)		85.3 (44.4)	
Outcome						
5-yr recurrence rate (%)	94/426 (22.1%)		52/243 (21.4%)		42/183 (23.0%)	
5-yr survival rate (%)	367/432 (85.0%)		205/247 (83.0%)		162/185 (87.6%)	

Abbreviation: SD, Standard deviation; *Others include serous mixed mucinous, serous mixed clear cell, mucinous mixed endometrioid, mucinous mixed clear cell, endometrioid mixed clear cell, poorly-differentiated, transitional, hepatoid, and small and squamous cell carcinoma. #All of the clear cell cases were regarded as grade 3; CP, Cyclophosphamide + Platinum; PT, Paclitaxel + Platinum; yr, year.
